# Enrichment of Terbium(III) under synergistic effect of biosorption and biomineralization by *Bacillus* sp. DW015 and *Sporosarcina pasteurii*

**DOI:** 10.1128/spectrum.00760-24

**Published:** 2024-06-25

**Authors:** Zijun Bian, Wei Dong, Xi Li, Yuexin Song, Huihong Huang, Kemin Hong, Kaijian Hu

**Affiliations:** 1 Jiangxi Key Laboratory of Mining and Metallurgy Environmental Pollution Control, Ganzhou, China; 2 School of Resources and Environmental Engineering, Jiangxi University of Science and Technology, Ganzhou, China; 3 Yichun Lithium New Energy Industry Research Institute, Jiangxi University of Science and Technology, Yichun, China; 4 School of Life Sciences, Jiangxi University of Science and Technology, Ganzhou, China; Dominican University New York, Orangeburg, New York, USA

**Keywords:** *Bacillus*, biosorption, biomineralization, Tb(III), microbially induced calcium carbonate precipitation (MICP)

## Abstract

Biosorption and biomineralization are commonly used for the immobilization of metal ions. Biosorption is commonly used as a green method to enrich rare earth ions from wastewater. However, little attention has been paid to the facilitating role of biomineralization in the enrichment of rare earth ions. In this study, a strain of *Bacillus* sp. DW015, isolated from ion adsorption type rare earth ores and a urease-producing strain *Sporosarcina pasteurii* were used to enrich rare earth elements (REEs) from an aqueous solution. The results indicate that biomineralization accelerates the enrichment of Terbium(III) compared to biosorption alone. Kinetic analysis suggests that the main mode of action of DW015 was biosorption, following pseudo-second-order kinetics (*R*^2^ = 0.998). The biomineralization of DW015 did not significantly contribute to the enrichment of Tb(III), whereas excessive biomineralization of *S. pasteurii* led to a decrease in the enrichment of Tb(III). A synergistic system of biosorption and biomineralization was established by combining the two bacteria, with the optimal mixed bacteria (*S. pasteurii*:DW015) ratio being 1:19. This study provides fundamental support for the synergistic effect of biosorption and biomineralization and offers a new reference for future microbial-based enrichment methods.

**IMPORTANCE:**

A weak microbially induced calcium carbonate precipitation (MICP) promotes the enrichment of Tb(III) by bacteria, while a strong MICP leads to the release of Tb(III). However, existing explanations cannot elucidate these mechanisms. In this study, the morphology of the bioprecipitation and the degree of Tb(III) enrichment were analyzed by X-ray diffraction (XRD), scanning electron microscopy (SEM), and energy dispersive spectroscopy (EDS). The data revealed that MICP could drive stable attachment of Tb(III) onto the cell surface, forming a Tb-CaCO_3_ mixed solid phase. Excessive rapid rate of calcite generation could disrupt the Tb(III) adsorption equilibrium, leading to the release of Tb(III). Therefore, in order for Tb(III) to be stably embedded in calcite, it is necessary to have a sufficient number of adsorption sites on the bacteria and to regulate the rate of MICP. This study provides theoretical support for the process design of MICP for the enrichment of rare earth ions.

## INTRODUCTION

Ionic rare earths, discovered in Ganzhou, China in 1969, are a kind of rare earths adsorbed in clay minerals in an ionic state. They possess the advantages of complete allotment, high content of medium and heavy rare earth elements, low radioactivity, and easy mining (1, 2). These properties render them valuable for high-tech industries and national defense industries (3, 4). However, in the leaching and mining of rare earths, a large amount of waste liquid is generated causing eutrophication of water (5, 6). The surrounding soil and rivers are infiltrated with residual rare earth ions in the smelting wastewater, resulting in wasted resources and a certain negative impact on the flora, fauna, and body of the environment (7, 8). Therefore, green and efficient technologies are urgently needed to meet the requirements of sustainable development of ionic rare earths.

A large number of physical and chemical recovery techniques related to rare earths have been reported both domestically and internationally. Some of the cutting-edge technologies include employing magnesium salt-leaching agents to leach rare earth ions instead of ammonium salt-leaching agents to reduce environmental pollution from ammonia–nitrogen wastewater and high-salinity sodium salt wastewater (5). In addition, modified adsorbents are prepared for the selective recovery of rare earths from rare earth wastewater (9). Zhang et al. synthesized two new recyclable rare earth ion adsorbents, covalent organic framework-p-phenylenediamine-cyanuric chloride, and covalent organic framework-melamine-cyanuric chloride via a one-step solvothermal method. These adsorbents exhibit adsorption capacities up to 150.88 and 168.19 mg/g at pH 5.5 and *t* = 35°C, respectively (10). However, these traditional recovery processes are more complicated and cause more rare earth ions loss. Because of the use of large amounts of electrical energy, inorganic leaching agents, organic extractants, and other chemical substances, high energy consumption, secondary pollution caused by the discharge of waste liquids, low recovery rate, and other problems are commonly observed (11).

The cells and spores of *Bacillus* species have also shown efficiency in the adsorption of rare earth ions due to their special structure with multiple phosphate groups (12, 13). Cheng et al. used *Bacillus licheniformis* to adsorb lanthanum in contaminated water with a maximum adsorption capacity of 113.98 mg/g and enrichment efficiency of up to 97.65% (14). The adsorption process of some microorganisms is usually accompanied by various biochemical reactions such as biomineralization (15). Lu et al. found that the presence of microbial mineralization doubled the recovery of rare earth ions compared with biosorption alone (16). The formation of minerals is directly or indirectly mediated by interactions between microorganisms and heavy metals, thereby affecting the transport and precipitation of metal ions (17). Biomineralization alters the local environment, creating conditions conducive to adsorption, thus highlighting the unique synergistic effect of biosorption and biomineralization.

Biomineralization is a process of forming complex products of interaction between inorganic mineral ions and organic compound molecules of organisms after undergoing nucleation, growth, and phase change (17). The more recent application is MICP based on urea hydrolysis, where urea hydrolysis is catalyzed by microbially produced urease to produce carbonate or complex salt crystals. Through this mechanism, metal cations in the environment can be co-precipitated with calcium ions, forming carbonate precipitation or other stable complexes (18–20). *Sporosarcina pasteurii*, *Sporosarcina aquimarina*, and *Bacillus megaterium* are popular urease-producing bacteria used to induce carbonate precipitation (21, 22). It is often applied in fracture repair, geotechnical impermeability sealing, and heavy metal pollution treatment (19, 23, 24). The calcium carbonate crystals produced by the mineralization reaction increase adsorption sites, encapsulating metal ions on the sites along with mineral coverage (16). Additionally, the utilization of the special surface structure of *Bacillus* and the alkaline pH conditions generated by growth metabolism fulfill the reaction requirements of MICP (25). However, there are few studies on the recovery of rare earth ions using MICP, the effect of MICP on microbial adsorption is unknown, and the strains that meet the enrichment are scarce. Therefore, it is necessary to screen suitable bacteria, establish and improve the synergistic enrichment system, and further study the enrichment of rare earth ions by biosorption and biomineralization.

In this study, Tb(III), a typical study object of REEs, is selected as the enrichment object. *Bacillus* strains that are tolerant against high-concentration of rare earth ions and produce urease were isolated. *S. pasteurii* with high urease activity was used as a control (26, 27). The enrichment performance of two bacteria for Tb(III) was studied, and the enrichment process was determined by an adsorption kinetic analysis. A more complete set of biomineralization and adsorption systems was continuously optimized and summarized. Finally, the study characterized the mineralization products and formation processes on the bacterial surface via X-ray diffraction (XRD), scanning electron microscopy (SEM), and energy dispersive spectroscopy (EDS) to elucidate the biosorption and biomineralization mechanisms.

## RESULTS AND DISCUSSION

### Isolation and identification of urease-producing *Bacillus* strain

A strain-producing urease named DW015 was obtained from the ionic rare earth mine. The colonies on the plates were smooth and white granules, which could change the urea agar medium from yellow to red. Initial experiments determined its urease activity as a follow-up experimental strain.

**FIG 1:**
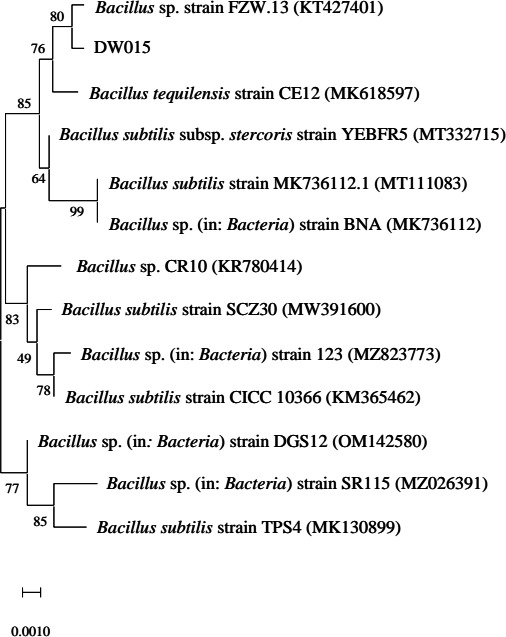
Phylogenetic tree of strain DW015 based on the 16S rRNA gene sequencing.

The 16S rRNA gene sequencing results of strain DW015 were submitted to NCBI, and a Blast search was conducted to compare them with known nucleic acid sequences. The phylogenetic tree of the strain was constructed by MEGA 11 (Fig. 1). The strain was identified as *Bacillus* sp. DW015, which is now conserved in the Guangdong Microbial Strain Conservation Center (GDMCC) with the accession number GDMCC No: 62949.

### Tolerance of bacterial strains against Tb(III)

**FIG 2:**
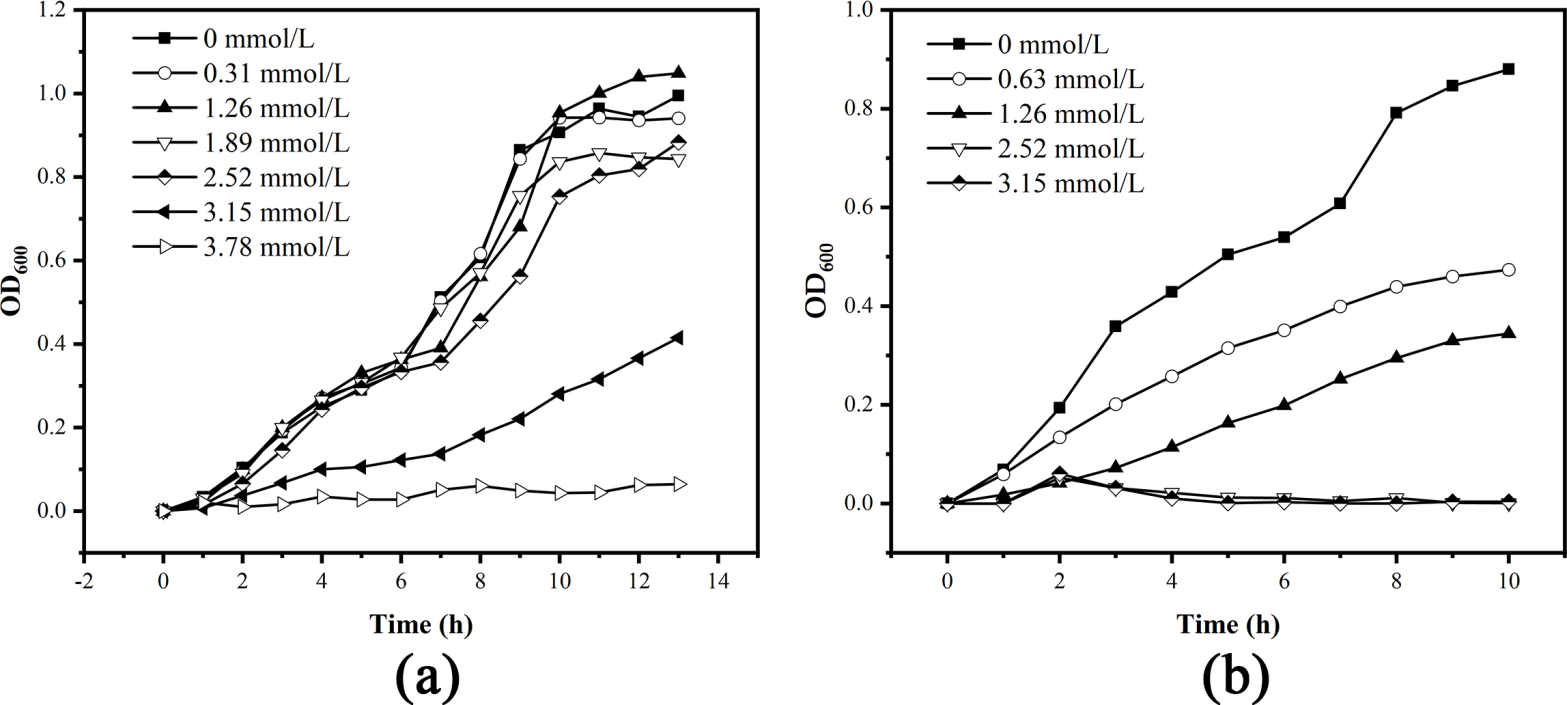
The growth curve at different Tb(III) concentrations of DW015 (a) and *S. pasteurii* (b).

The growth curves at different Tb(III) concentrations of DW015 and *S. pasteurii* were presented in Fig. 2. When the Tb(III) concentration was below 1.26 mmol/L, Tb(III) showed almost no response to the growth of DW015 (Fig. 2a). As the Tb(III) concentration increased, the growth of DW015 was progressively inhibited. The growth was completely stopped at a concentration of 3.78 mmol/L. The semi-lethal concentration of DW015 was 3.09 mmol/L. Tb(III) had a strong inhibitory effect on the growth of *S. pasteurii* and completely stopped growth at a concentration of 2.52 mmol/L, with a semi-lethal concentration of 1.53 mmol/L (Fig. 2b). These findings suggest that DW015 isolated from the abandoned rare earth mines had stronger adaptability to rare earth ions than *S. pasteurii*.

Rare earth elements cause toxicity to cells, involving lipid peroxidation along with changes in membrane permeability and polarization (28). Therefore, the toxicity of Tb(III) inevitably causes bacterial death. Dead cells were found to be unable to induce mineralization and exhibited significantly reduced adsorption capacity for metal ions compared to that of living cells (29, 30). DW015 had stronger resistance to rare earth ions than *S. pasteurii* and better maintained its activity to ensure effective adsorption and mineralization during the enrichment of rare earth ions.

### Adsorption and mineralization of Tb(III) by *Bacillus* sp. DW015

**FIG 3:**
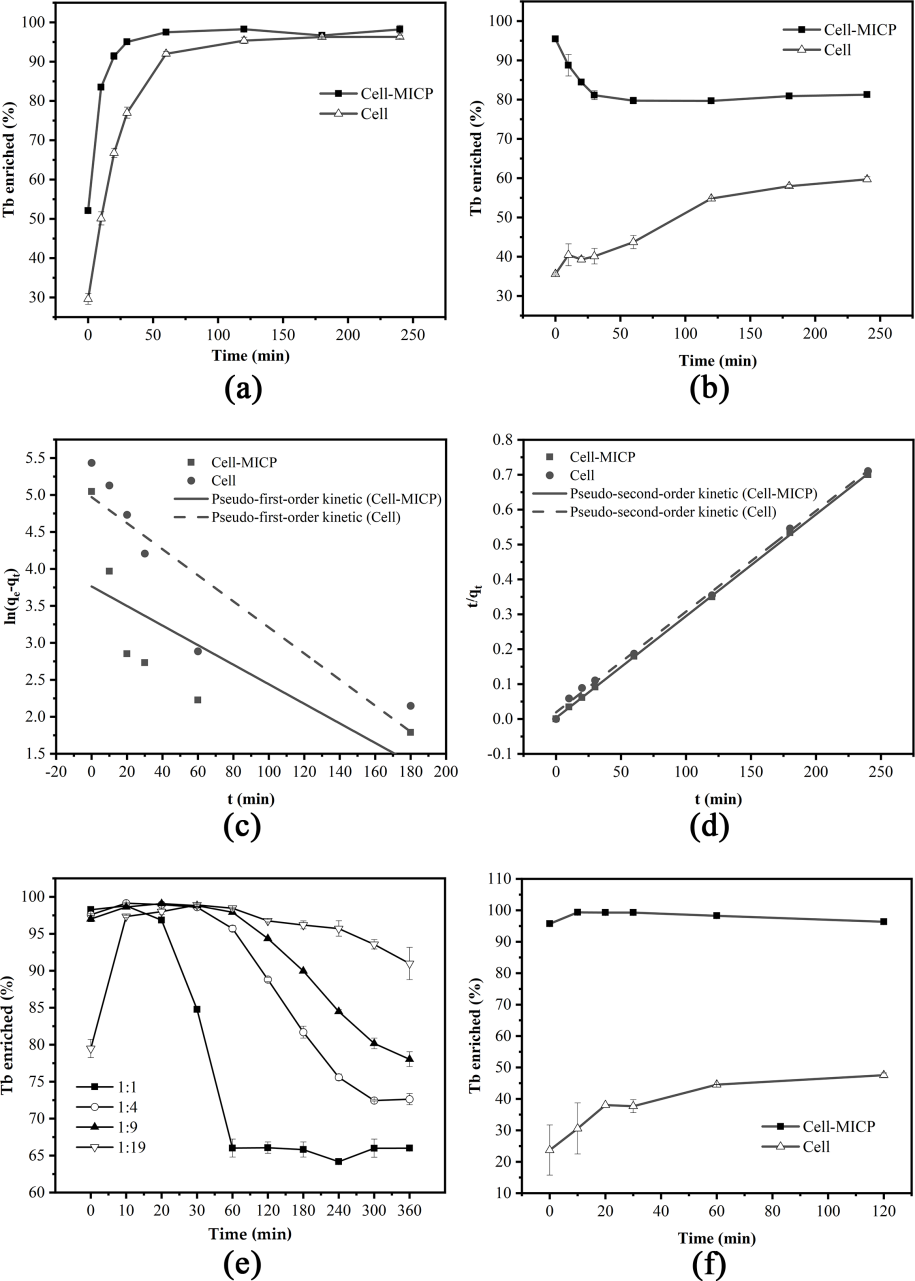
Enrichment of Tb(III) from aqueous solution. (a) Tb(III) enrichment by strain DW015. (b) Tb(III) enrichment by strain *S. pasteurii*. (c) Pseudo-first-order kinetic model fitting. (d) Pseudo-second-order kinetic model fitting. (e) Enrichment of Tb (III) with different rations of *S.pasteurii* and DW015. (f) Enrichment of Tb(III) with *S. pasteurii*:DW015 = 1:19 (total OD_600_ = 0.5). The initial Tb(III) concentration of 400 µmol/L was approximately the concentration of rare earth ions in the wastewater. Two types of enrichment: cellular adsorption (Cell) and enrichment with MICP (Cell-MICP).

The effect of cellular adsorption and adsorption with MICP on the enrichment of Tb(III) from solution was investigated in DW015 with initial concentrations of Tb(III) at 400 µmol/L under the conditions of OD_600_ = 1, pH 7.4, adsorption time of 4 h, and temperature of 37°C. When the initial concentration of Tb(III) was 400 µmol/L, the enrichment efficiency via adsorption and MICP increased by 19.1% compared to cellular adsorption from 0 to 1 h (Fig. 3a). The enrichment efficiency of DW015 increased rapidly from 0 to 1 h with increasing contact time, especially at the first contact. The binding sites such as the first exposed groups on the cell surface were utilized instantaneously, leading to a sharp increase in the enrichment efficiency. The enrichment efficiency slowly increased and reached equilibrium after 1 h, with a maximum enrichment efficiency of about 98%. The results showed *Bacillus* sp. DW015 had a strong adsorption of Tb(III).

The mechanism of MICP for metal ion enrichment has been described in many studies (17, 31, 32). One explanation suggests that the presence of MICP leads to the coverage of the cell surface with induced calcium carbonate, thereby augmenting the cell-specific surface area to achieve an increase in the Tb(III) adsorption sites (16, 33). Consequently, a further increase in the rate of Tb(III) enrichment was achieved. The CO_3_^2-^ of this experiment was obtained mainly through the dissolution of CO_2_ in air in an alkaline solution, which was a very slow process (34). Due to the failure to produce significant carbonate precipitation, the exact mechanism for the weak MICP of DW015 to enhance the rate of Tb(III) enrichment was still debatable. Therefore, the mineralization system needs to be further optimized.

### Adsorption and mineralization of Tb(III) by *S. pasteurii*

In order to better explore the effect of MICP on the enrichment of Tb(III), *S. pasteurii* with high urease activity was applied to treat 400 µmol/L Tb(III). It was clear that *Bacillus* sp. DW015 had stronger adsorption of Terbium but weaker mineralization than *S. pasteurii* (Fig. 3a and b). *S. pasteurii* demonstrated limited Tb(III) adsorption capability, with the enrichment efficiency gradually increasing and stabilizing within 0–2 h, reaching a maximum enrichment efficiency of 59.7% (Fig. 3b). Unlike cellular adsorption, where the enrichment efficiency was only 35.6% at the instant of contact, MICP in *S. pasteurii* led to a significant enhancement in the Tb(III) enrichment efficiency, reaching 95.4% upon contact initiation. This exhibited a completely different trend from the cellular adsorption curve. The highest enrichment efficiency was reached at the instant of contact, followed by a slow decrease over time. It gradually reached the equilibrium at 1 h and the Tb(III) enrichment efficiency remained at about 80%. When dilute hydrochloric acid was added dropwise to the precipitate excluding the supernatant, the precipitate showed noticeable bubbles. It was thus clear that the enrichment of rare earth ions by *S. pasteurii* was mainly attributed to MICP within a short period of contact.

### Adsorption kinetic model of Tb(III) by *Bacillus* sp. DW015

From the correlation coefficients (*R*^2^) of the fitted results (Table 1) and the fitted graphs (Fig. 3c and d), the fitted results of the adsorption process of Tb(III) by both modes of action of DW015 were poorer than those of the pseudo-first-order kinetic model, with *R*^2^ values of 0.543 and 0.816, respectively. The *R*^2^ values of the pseudo-second-order kinetic model fitted by the kinetic model were all >0.99, and the fitted adsorption amount did not differ much from the actual equilibrium adsorption amount derived from the experiment, indicating that the adsorption of Tb(III) by both modes of action of *Bacillus* sp. DW015 was more consistent with the pseudo-second-order kinetic model. *q_t_* = 1/(0.0032*/t* + 0.00292) is the adsorption amount of DW015 adsorption with MICP at *t*, while the adsorption amount of DW015 cellular adsorption at *t* is *q_t_* = 1/(0.0191*/t* + 0.00289).

**TABLE 1:** Fitting parameters of adsorption kinetic model of DW015 to Tb(III)

Modes of action	Amount adsorbed (µmol/L)	Pseudo-first-order kinetic			Pseudo-second-order kinetic		
		*K*_1_	*q_e_*/(µmol/L)	*R* ^2^	*K*_2_	*q_e_*/(µmol/L)	*R* ^2^
Cell-MICP	343.284	−0.013	43.077	0.543	0.003	342.466	1.000
Cell	338.273	−0.018	144.171	0.816	0.001	346.021	0.998

The adsorption process of biosorbents mostly conformed to the pseudo-second-order kinetic model, and the two adsorption modes of DW015 aligned more closely with the pseudo-second-order kinetic model, which was consistent with the experimental results (35, 36). According to some previous studies, it is known that the adsorption of Tb(III) by DW015 is mainly chemisorption, where the mass transfer resistance occurs in the outer cell layer, and the adsorption depends on the action of various groups on the cell surface, forming chemical bonds stronger than physical adsorption (13, 15, 37). Under nutrient-deficient conditions, bacteria are unable to provide sufficient CO_3_^2-^ and maintain an alkaline pH to induce effective mineralization reactions (25, 29). The MICP in the “urea-Ca(II)” mineralization exhibits relatively weak effects on enhancing the efficiency of Tb(III) enrichment. Therefore, chemisorption on the cell surface plays a dominant role in the enrichment of Tb(III) by DW015.

### Optimization of a method for Tb(III) enrichment by *Bacillus* sp. DW015 and *S. pasteurii*

In order to enhance the mineralization capacity of the present enrichment system, the study made full use of the strong mineralization capacity of *S. pasteurii*. The mineralization and adsorption experiments were carried out by adding DW015 and *S. pasteurii* to the “urea-Ca(II) mineralization system” in a certain ratio (total OD_600_ = 1). This solved the low mineralization efficiency of DW015 and ensured the accuracy of the experiments.

When *S. pasteurii*:DW015 = 1:1 (Fig. 3e), the Tb(III) enrichment efficiency remained stable at about 96% from 0 to 20 min, with an additional stabilization period compared with the mineralization of *S. pasteurii* alone (Fig. 3b). However, the Tb(III) enrichment efficiency began to decrease with time after 20 min and finally stabilized at 66% after 60 min. The Tb(III) enrichment efficiency decreased by 14% during the equilibrium phase compared to the mineralization of *S. pasteurii* alone. When *S. pasteurii*:DW015 = 1:4, the Tb(III) enrichment efficiency stabilized at 98% from 0 to 30 min, and then decreased rapidly after 30 min. When *S. pasteurii*:DW015 = 1:9, the Tb(III) enrichment efficiency was maintained at 98% from 0 to 60 min and then gradually decreased at a rate significantly smaller than the former. When *S. pasteurii*:DW015 = 1:19, the Tb(III) enrichment efficiency showed a brief upward trend from 0 to 10 min to reach 97% rapidly and maintain equilibrium. Although Tb(III) enrichment efficiency also showed a decreasing trend after 1 h, the enrichment efficiency remained at a high level from 1 to 6 h above 90%. It is thus clear that a lower proportion of MICP induced by *S. pasteurii* is sufficient to drive the attachment of Tb(III) on the cell surface, and a higher proportion of DW015 favors stable Tb(III) attachment on the cell surface. The efficient and stable enrichment of Tb(III) can be satisfied by the mixture of *S. pasteurii*:DW015 = 1:19.

*Bacillus*, negatively charged various functional groups, mucilage, structures, and groups on the cell surface, can be used as an efficient and sustainable biosorbent for rare earth ions (29, 38). When the pH in the environment rises, the ionization balance of the functional groups on the cell surface is altered and deprotonated, increasing its affinity for metal cations and enhancing adsorption (39). Thus, a higher abundance of these functional groups implies a greater affinity for rare earth ions. This suggests that DW015 has more functional groups than *S. pasteurii*. Additionally, the functional groups have an important contribution to the adsorption of rare earth ions when the pH is adjusted by MICP.

Due to the remarkable mineralization ability of *S. pasteurii*, the addition of 5% *S*. *pasteurii* to DW015 (*S. pasteurii*:DW015 = 1:19) could compensate for the system deficiency in the difficult MICP reaction. When a rapid increase in the enrichment rate occurred (*t* = 0–10 min), the Tb(III) enrichment efficiency could reach 97% within 10 min. After a period of equilibrium, the enrichment efficiency slowly decreased but stabilized over 90% within 6 h. However, the adsorption efficiency of DW015 could also reach 98% in the previous experiments. When the bacterial concentration was reduced to half (*S. pasteurii*:DW015 = 1:19 and total OD_600_ = 0.5, Fig. 3f), the cell adsorption efficiency was substantially weakened, with the highest efficiency still not reaching 50%. In contrast, the enrichment efficiency of the “Cell-MICP” remained high, reaching up to 99%. This shows that the promotion of Tb(III) adsorption by cells with MICP is very significant.

### XRD analysis of *Bacillus* before and after mineralization and adsorption

**FIG 4:**
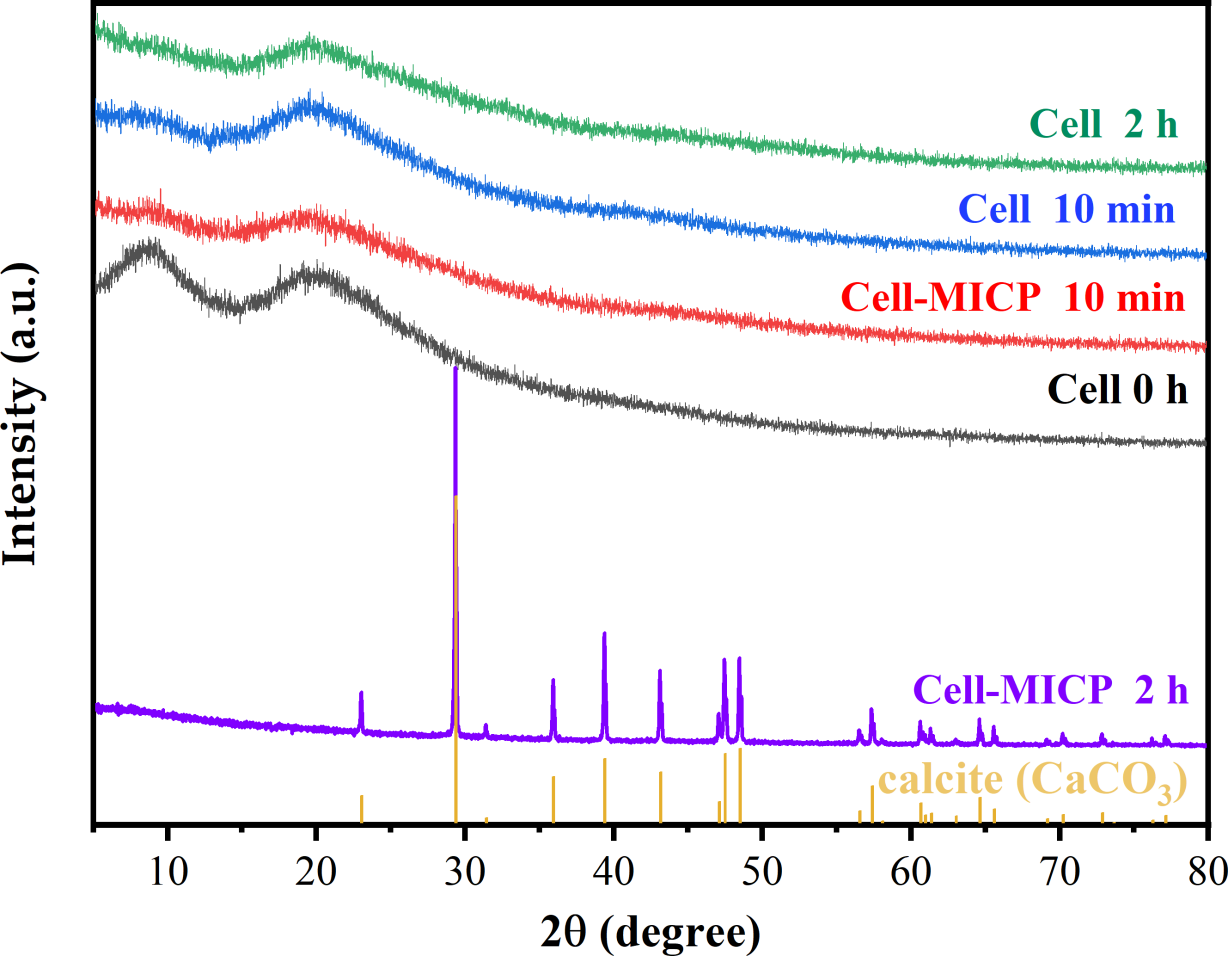
XRD patterns of mixed bacteria (*S. pasteurii*:DW015 = 1:19) before and after adsorption and mineralization of Tb(III). Two types of enrichment: cellular adsorption (Cell) and enrichment with MICP (Cell-MICP).

To investigate the chemical properties and precipitation morphology before and after the interaction between bacteria and Tb(III), XRD analysis was performed before and after adsorption and mineralization of Tb(III) by mixed bacteria (*S. pasteurii*:DW015 = 1:19) (Fig. 4). CaCO_3_ was formed gradually with increasing contact time (40). The diffraction peaks observed on the XRD spectra after 2 h of bacterial interaction with Tb(III) using MICP could be well matched with calcite (PDF#47-1743), indicating that the strain-induced mineralization to form calcium carbonate precipitates. Besides, no obvious sharp diffraction peaks were detected on the XRD pattern, indicating that Tb(III) did not form minerals during the cell adsorption and initial period of MICP. Existing studies have shown that only a small portion of the rare earth ions combine with other anions (e.g. oxalate) to precipitate as compounds (18, 41, 42). The results did not reveal any compounds formed by Tb(III).

### SEM-EDS analysis of *Bacillus* before and after mineralization and adsorption

**FIG 5:**
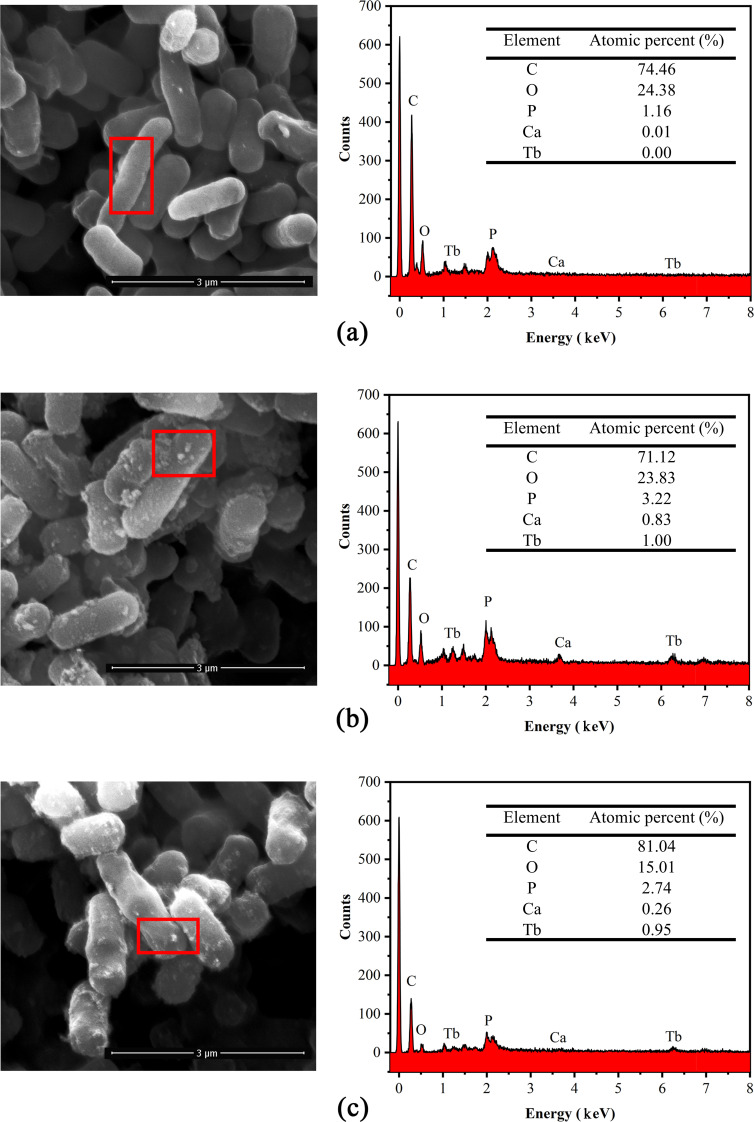
SEM images and EDS spectra of mixed bacteria (*S. pasteurii*:DW015 = 1:19) before and after adsorption and mineralization of Tb(III). P peak covered by Au. Two types of enrichment: cellular adsorption (Cell) and enrichment with MICP (Cell-MICP). (a) Cell, *t* = 0 min; (b) Cell-MICP, *t* = 10 min; (c) Cell, *t* = 120 min.

**FIG 6:**
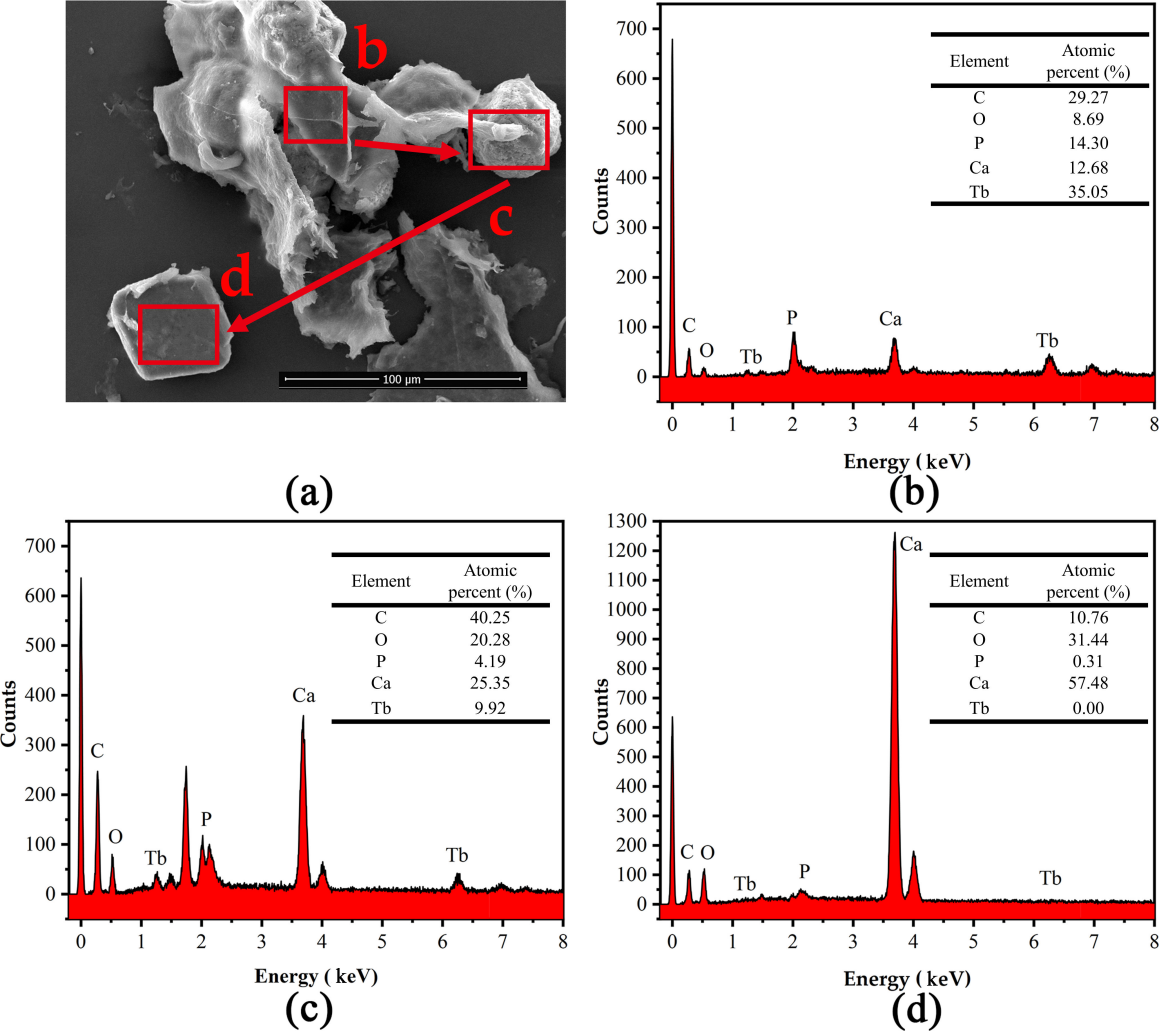
(a) SEM images of mixed bacteria (*S. pasteurii*:DW015 = 1:19) of mineralization when the time is 2 h. EDS spectra of (b) amorphousness, (c) sphere, (d) calcite.

The precipitates of cell mineralization and adsorption were examined by SEM at two representative stages (*t* = 10, 120 min). The results of EDS analysis of the elemental signal on the cell surface at each stage are presented in Fig. 5 and 6. Before the interaction with Tb(III), cells were cylindrical in shape, with a smooth surface (Fig. 5a). After 10 min of adsorption with MICP, bright irregular attachments appeared on the cell surface. The ratio of C, Ca, and Tb increased, indicating that Tb(III) started to be adsorbed while inducing the production of CaCO_3_ (Fig. 5b). No minerals were formed on the cell surface before or after the adsorption of cells, with irregular attachments present on their surfaces (Fig. 5c). The proportion of Tb element increased with the increase of adsorption time, which is consistent with the results of existing studies (13, 22, 26). While the two cases exhibit similarities, there is a large difference in the time required to reach high adsorption rates. Hence, it can be inferred that MICP played an important role in the enrichment process during the period.

The surface morphology and the energy spectra of the three corresponding positions of Tb(III) adsorbed by bacteria under the action of MICP were investigated and shown in Fig. 5. When the reaction reached 2 h, MICP induced the production of a large number of squares and a small number of spheres, wrapping the bacteria. As per the XRD results, these square minerals are identified as calcite. The signals of Ca and Tb were greatly enhanced, indicating that the bacteria induced the formation of a large number of CaCO_3_ crystals (Fig. 6a and b). During this period, the cells and the adsorption sites on their surface were almost completely encapsulated. From the Ca signals at the three positions, it can be seen that the proportion of Ca in “b-c-d” was increasing (i.e., the crystalline growth process of CaCO_3_ minerals produced by MICP was from amorphous to spherical aragonite and finally to massive calcite) (22, 43). The atomic ratio C:O = 1:3 in the lumpy material was identified as CaCO_3_ (Fig. 6d).

The proportion of Tb decreased continuously during the growth of CaCO_3_ minerals, and a portion of Tb(III) located at the mineralization site was released by Ca(II) exchange. This resulted in a weakened Tb signal, with the vast majority of the Tb signal being masked by the calcium carbonate generated during MICP (16). Therefore, it can be presumed that the vast majority of Tb is encapsulated in calcite after the calcite is formed. This is the reason why the enrichment efficiency of Tb(III) remains high, even when the adsorption sites are masked. It is clear that, from SEM-EDS and XRD results, stronger Tb signals can be detected on the amorphous minerals formed on the cell surface. It further demonstrates similar results to Lu’s results (16), that Ca and Tb exist as a Tb-CaCO_3_ mixture phase in the initial period of the MICP stage and are gradually encapsulated by the subsequently generated calcite.

### Tb(III) enrichment processes of *Bacillus* with MICP

It has been shown that microorganisms regulate metal ion enrichment and precipitate (44). Microorganisms provide a large number of active sites for adsorption and mineralization, and MICP reduces the activation energy required for the precipitation of metal ions (29). Intuitively, MICP can drive metal ions [Ca(II) and Tb(III)] toward the nucleation sites, reaching a “supersaturated state” at the cell surface, thus, significantly enhancing the Tb(III) enrichment efficiency. However, as the mineralization reaction proceeds, the nucleation sites become gradually covered by CaCO_3_ crystals, and a part of Tb(III) is gradually released into the solution, making the adsorption curve more similar to the release curve. The other part of Tb(III) was encapsulated into CaCO_3_ crystals until the formation of stable minerals, at which point no more Tb(III) was released, as evidenced by the curve reaching equilibrium (Fig. 3b). No similar results and reasonable explanations were found in various literature reports. Therefore, it is presumed that the effect of the MICP in increasing the rate of Tb(III) enrichment occurs mainly in the initial period of reaction or even at the moment of contact.

The mineral growth process of MICP is very similar to the sequestration process of Tb. The overlay of CaCO_3_ inevitably displaces the original Tb(III) sites and disrupts the cellular adsorption equilibrium (45). This also explains why the enrichment efficiency of Tb(III) decreased afterward (Fig. 3e). However, as the adsorption capacity of DW015 for Tb(III) is much greater than that of *S. pasteurii*, the numerous functional groups (carboxyl, hydroxyl, phosphate groups, etc.) on the surface of the bacteria also contribute to the formation of stable calcite. Therefore, Tb(III) will not be easily released but will be encapsulated in the gradually formed mineral (22). It serves a good stabilizing role during the precipitation process.

**FIG 7:**
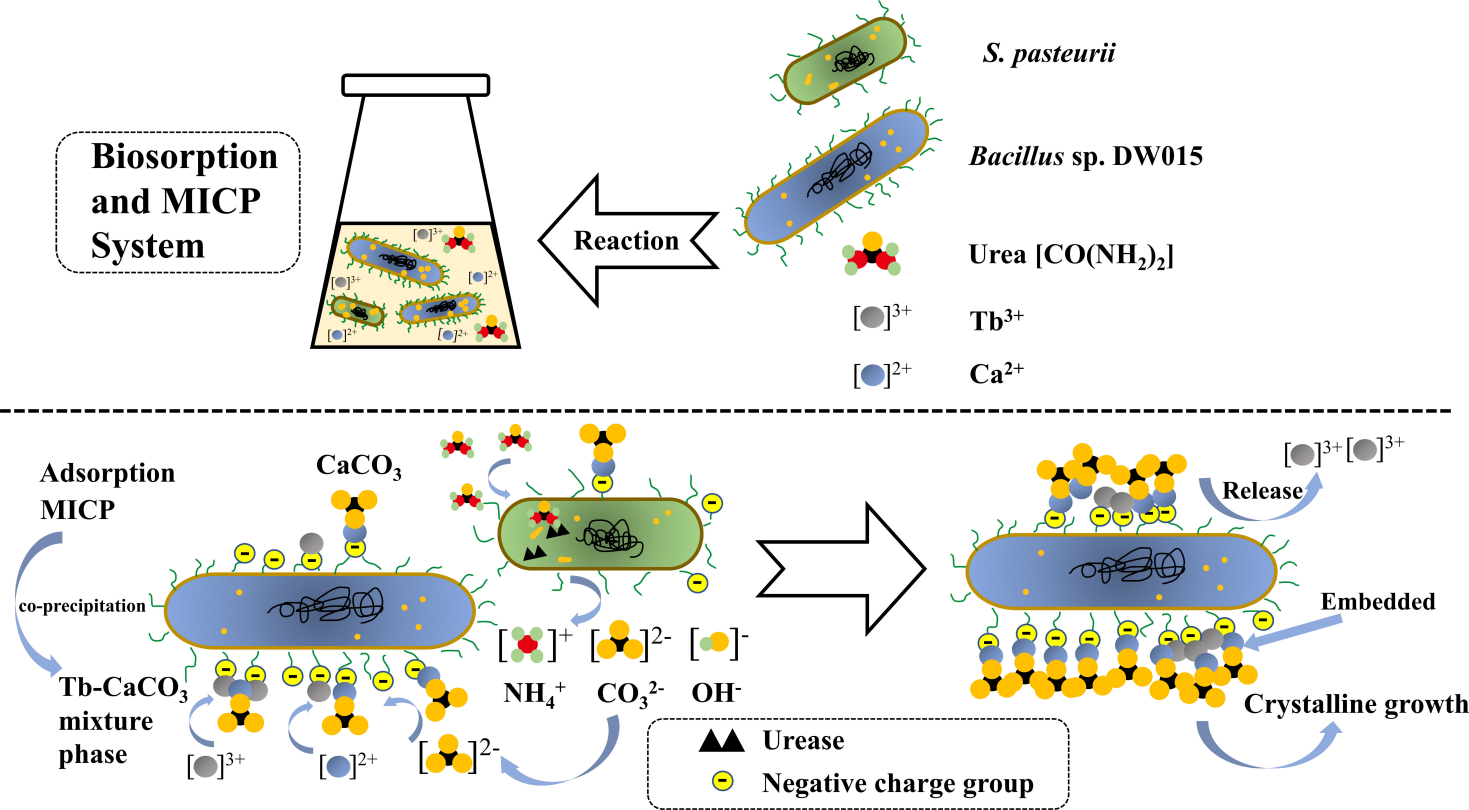
Schematic diagram of Tb(III) enrichment processes by *Bacillus* sp. DW015 and *S. pasteurii*.

As shown in Fig. 7, the final putative Tb(III) enrichment processes were (i) Chemisorption and MICP sites that were provided by DW015. The CO_3_^2-^ required for MICP was provided by *S. pasteurii*, increasing the ambient pH and inducing Ca(II) binding to the cell surface; (ii) The “supersaturated state” formed by MICP co-precipitates with Ca to promote rapid attachment of Tb(III) to the surface. At this stage, Tb(III) was precipitated through the combined action of chemisorption and MICP. Free Tb(III) was basically adsorbed completely and CaCO_3_ began to form on the cell surface; (iii) As CaCO_3_ precipitation continued to increase, vaterite and calcite began to form, and the vast majority of Tb(III) was embedded into CaCO_3_.

This work shows that the high Tb(III) enrichment efficiency (98%) can be achieved by *Bacillus* sp. DW015 within 30 min, which is mainly attributed to chemisorption. Enhancing MICP by supplementation with 5% *S*. *pasteurii* can shorten the enrichment time to 10 min or even shorter. Ca(II) and Tb(III) are driven by MICP to nucleation sites on the cell surface, which enhances the enrichment efficiency of Tb(III) during the initial period of crystal production of CaCO_3_. The combined adsorption-mineralization recovery method can be developed as a promising method for enriching rare earth resources.

## MATERIALS AND METHODS

### Isolation of urease-producing microorganisms

The dilution plate method was used to isolate strains from abandoned rare earth mines’ soil samples. Strains were inoculated from glycerol tubes onto LB solid medium (10 g peptone, 5 g yeast extract, 10 g NaCl, 20 g agar, and 1,000 mL deionized water) and incubated for 12 h. The activated single colony was selected and inoculated into a urease screening medium and incubated at 37°C for 24 h. Urea agar medium consisted of 2 g potassium dihydrogen phosphate, 5 g sodium chloride, 1 g peptone, 4 mL 0.2% phenol red, 20 g agar, and 890 mL water. It was autoclaved and then cooled to about 50°C. Then 100 mL 20% urea solution and 10 mL 10% glucose solution were filtered and sterilized before being added.

Urea is hydrolyzed by urease-producing bacteria to form NH_4_^+^ and HCO_3_^-^. An increase in pH in the medium causes the phenol red indicator to turn from yellow to red (32, 46). In this way, the eligible strains were screened.

### Identification of *Bacillus* strain based on 16S rRNA gene sequencing

The screened *Bacillus* strains were cultured in LB liquid and collected when they grew to the logarithmic phase. They were then sent to Sangon Biotech (Shanghai) Co., Ltd. for 16S rRNA gene sequencing. After the products were sequenced, the sequences measured were input to NCBI for comparison, and the phylogenetic tree was constructed using *MEGA 11*.

### Preparation of *S. pasteurii* culture

*S. pasteurii* (ATCC11859) was purchased from the Beijing Biological Conservation Center. To activate and recover the strain, CASO medium was prepared with the following composition: 15 g tryptone, 5 g soy peptone, 5 g NaCl, and 900 mL deionized water with pH adjusted to 7.3. It was then autoclaved and cooled to about 50°C before filtering and removing bacteria by adding 100 mL of 20% urea solution.

### Analysis of tolerance against Tb(III)

Single colonies of *Bacillus* were transferred to an LB liquid medium with terbium ion concentrations of 50, 300, 400, 500, 600, and 900 mg/L. They were incubated in a shaker for 12 h at 37°C and with a speed of 180 rpm. The OD_600_ of the bacterial solution was measured by hour, and curves were plotted by *Origin 2021*.

The survival rate of cells was calculated from the bacterial concentrations at equilibrium. The survival rate and the related Tb^3+^ concentration were fitted with the logistic function in *Origin 2021*, with the initial value parameter and final value parameter set to 1 and 0, respectively. The equations of cell survival rate and Tb^3+^ concentration were derived. The semi-lethal concentration was the Tb^3+^ concentration when the survival rate was 50%.

### Determination of rare-earth ion Tb(III)

The rare earth ion Tb(III) can be combined with excess 2,6-pyridine dicarboxylic acid (DPA) to form a complex. It fluoresces at a specific wavelength, and its fluorescence intensity is significantly linear with the concentration of rare earth ions in a certain range (12, 13). The fluorescence intensity was measured with the excitation wavelength of 275 nm and the emission wavelength of 545 nm by adding 100 µL of 100 mmol/L HEPES buffer and 10 µL of 10 mmol/L DPA solution to 90 µL solution. The results were measured in a 96-well enzyme linked immunosorbent assay (ELISA) plate, and the concentration of rare earth ions in the solution was calculated by *Origin 2021*.

### Adsorption and mineralization of Tb(III) by bacterial strains

The bacterial broth cultured to the logarithmic phase was centrifuged at 4,000 rpm. Following centrifugation, the supernatant was removed, washed twice with sterile water, and finally diluted with sterile saline to obtain OD_600_ = 2. The cytosol (OD_600_ = 1 after mixing) was mixed with a solution containing Tb(III) with different concentrations (100, 200, 400 µmol/L), 25 mmol/L CaCl_2_, and 2% urea. The volume of the solution was 50 mL and the pH was adjusted to 7 with NaOH and HCl (19, 40). They were incubated in a shaker at 37°C and with a speed of 180 rpm. CaCl_2_ was chosen as the calcium source for the inhibitory effect of nitrate on urease activity (23). After mineralization, the cytosol loaded with rare earth ions was obtained by centrifugation at 10,000 rpm for 2 min at regular intervals, with *Bacillus* serving as a control bacterium (40). The experimental groups were set up (as shown in Table 2) (47). Each experiment was repeated three times, and hydrochloric acid was added dropwise to the reacted precipitate to confirm the occurrence of a mineralization reaction in bacteria. The concentration of rare earth ions in the supernatant was measured after centrifugation, and its calculation formula is shown in Equation (1):


(1) $$R\;=\;\left(1\;-\;c/c_{0}\right)\;\times \;100\%$$

**TABLE 2:** Mineralization and adsorption experiments

	Cell	Ca(II), urea	Tb(III)
Experimental group	+^*a*^	+	+
Control group	+	−^*b*^	+
Blank group I	−	+	+
Blank group II	**−**	−	+

^*a*^ Material added into the system.

^*b*^ Material not added into the system.

Where *R* is the enrichment efficiency of rare earth ions, *c_0_* is the concentration of rare earth ions in the solution before adsorption (µmol/L), and *c* is the concentration of rare earth ions in the solution after adsorption (µmol/L).

Similar adsorption experiments have been conducted to study the enrichment efficiency by directly inoculating bacteria into the medium containing the target removers (48, 49). However, a direct addition of Tb(III) to the medium proved unfeasible. Excessive medium interference with Tb(III) was considered, so the factor of the medium must be excluded to ensure the accuracy of the experiment.

### Adsorption kinetic model of Tb(III) by bacterial strains

The relationship between adsorption amount and adsorption time was obtained through adsorption experiments, and the kinetic model was fitted by *Origin 2021*. The pseudo-first-order Equation and pseudo-second-order Equation are shown in Equations (2) and (3) and 3, respectively,


(2) $$ln\;\left(q_{e}\;-\;q_{t}\right)\;=\;lnq_{e}\;-\;K_{1}t,$$

(3) $$t/q_{t}\;=\;1/K_{2}q_{e}^{2}\;+\;t/q_{e},$$

where *q_e_* and *q_t_* represent the amount (µmol/L) of Tb(III) adsorbed on bacteria at equilibrium and time *t*, respectively. *K*_1_ and *K*_2_ are pseudo-first-order and pseudo-second-order adsorption rate constants, respectively.

### Characterization of Tb(III) biosorption and biomineralization

The mixture of bacteria before and after the interaction was centrifuged (10,000 rmp, 10 min) and the precipitate was collected. The bacteria precipitates were washed three times with deionized water, followed by fixation using 2.5% glutaraldehyde solution. Subsequently, the precipitate was dehydrated with ethanol, first at a pre-cooled temperature of −20°C and then −80°C. Then, they were dried in a vacuum freeze dryer for 24 h. The samples were fully ground with a mortar and pestle to achieve a particle size of 300 mesh. The sample powder was collected for characterization.

The samples were sprayed with Au and observed by SEM (Philips, XL30 W/TMP, Holland). Additionally, the surface elemental composition was analyzed by EDS (Bruker, Esprit 1.9, Berlin, Germany). The composition of the precipitated phases was analyzed with an X-ray powder diffractometer (Bruker, D8 Advance, Karlsruhe, Germany) within the range of 5°–80° (2θ) for Cu Kα radiation basis.

## References

[1] Shen L, Zhou H, Shi Q, Meng X, Zhao Y, Qiu G, Zhang X, Yu H, He X, He H, Zhao H. 2023. Comparative chemical and non-contact bioleaching of ion-adsorption type rare earth ore using ammonium sulfate and metabolites of Aspergillus niger and Yarrowia lipolytica to rationalise the role of organic acids for sustainable processing. Hydrometallurgy 216:106019. doi:10.1016/j.hydromet.2023.106019

[2] Wang W, Xu Y, Yan R, Zhang Z. 2021. New insights into ion adsorption type rare-earths mining—bacterial adsorption of yttrium integrated with ammonia nitrogen removal by a fungus. Sustainability 13:9460. doi:10.3390/su13169460

[3] S-l H, Liu J. 2018. Exploition of ionic type rare-earth mineral of South Jiangxi and its influence on the environment. J Magn Mater Devices 49:61–63. doi:10.19594/j.cnki.09.19701.2018.03.016

[4] Brown RM, Mirkouei A, Reed D, Thompson V. 2023. Current nature-based biological practices for rare earth elements extraction and recovery: bioleaching and biosorption. Renewable Sustainable Energy Rev 173:113099. doi:10.1016/j.rser.2022.113099

[5] Xiao Y, Huang X, Feng Z, Liu X, Huang L, Long Z. 2015. Environmental impact assessment and prospect for ion-adsorption type rare Earths ore leaching by magnesium salt. J Chinese Soc Rare Earth 33:1–9. doi:10.11785/S1000-4343.20150101

[6] Zou Z, Yang H, Zhang S, Chi W, Wang X, Liu Z. 2022. Nitrogen removal performance and microbial community analysis of immobilized biological fillers in rare earth mine wastewater. Biochem Eng J 186:108559. doi:10.1016/j.bej.2022.108559

[7] Xia CF, Jin JC, Yuan L, Zhao J, Chen XY, Jiang FL, Qin CQ, Dai J, Liu Y. 2013. Microcalorimetric studies of the effect of cerium (Ⅲ) on isolated rice mitochondria fed by pyruvate. Chemosphere 91:1577–1582. doi:10.1016/j.chemosphere.2012.12.049 23352518

[8] Chen Z, Fei Y, Liu W-S, Ding K, Lu J, Cai X, Cui T, Tang Y-T, Wang S, Chao Y, Qiu R. 2022. Untangling microbial diversity and assembly patterns in rare earth element mine drainage in South China. Water Res 225:119172. doi:10.1016/j.watres.2022.119172 36191530

[9] Hu K, Liu Y, Zhou X, Hussain S, Li K, Chen Q, Zhang C, Song W, Li X, Wan Y. 2023. Highly selective recovery of rare earth elements from mine wastewater by modifying kaolin with phosphoric acid. Sep Purif Technol 309:123117. doi:10.1016/j.seppur.2023.123117

[10] Zhang Y, Liu D, Guo W, Ding Y. 2023. Less-precious nitrogen-rich covalent organic frameworks capable of effective rare earth recovery from water. J Mol Liq 372:121229. doi:10.1016/j.molliq.2023.121229

[11] Yu Z, Han H, Feng P, Zhao S, Zhou T, Kakade A, Kulshrestha S, Majeed S, Li X. 2020. Recent advances in the recovery of metals from waste through biological processes. Bioresour Technol 297:122416. doi:10.1016/j.biortech.2019.122416 31786035

[12] Dong W, Li S, Camilleri E, Korza G, Yankova M, King SM, Setlow P. 2019. Accumulation and release of rare Earth ions by spores of Bacillus species and the location of these ions in spores. Appl Environ Microbiol 85:e00956-19. doi:10.1128/AEM.00956-19 31253678 PMC6696969

[13] Dong W, Wang H, Ning Z, Hu K, Luo X. 2022. Bioadsorption of Terbium(III) by spores of Bacillus subtilis. Minerals 12:866. doi:10.3390/min12070866

[14] Cheng Y, Zhang L, Bian X, Zuo H, Dong H. 2018. Adsorption and mineralization of REE—lanthanum onto bacterial cell surface. Environ Sci Pollut Res 25:22334–22339. doi:10.1007/s11356-017-9691-0 28699006

[15] Gavrilescu M. 2022. Microbial recovery of critical metals from secondary sources. Bioresour Technol 344:126208. doi:10.1016/j.biortech.2021.126208 34715340

[16] Lu Y, Chen L, Yu Q, Cheng C, Cheng Y. 2021. Fusarium oxysporuminduces mineralization recovery rare earth ions Lanthanum (Ⅲ). Acta Microbiologica Sinica 61:1621–1631. doi:10.13343/j.cnki.wsxb.20210104

[17] Lin H, Zhou M, Li B, Dong Y. 2023. Mechanisms, application advances and future perspectives of microbial-induced heavy metal precipitation: a review. Int Biodeterior Biodegrad 178:105544. doi:10.1016/j.ibiod.2022.105544

[18] Cheng Y, Zhang T, Zhang L, Ke Z, Kovarik L, Dong H. 2022. Resource recovery: adsorption and biomineralization of cerium by Bacillus licheniformis. J Hazard Mater 426:127844. doi:10.1016/j.jhazmat.2021.127844 34838363

[19] Bhattacharya A, Naik SN, Khare SK. 2018. Harnessing the bio-mineralization ability of urease producing Serratia marcescens and Enterobacter cloacae EMB19 for remediation of heavy metal cadmium (II). J Environ Manage 215:143–152. doi:10.1016/j.jenvman.2018.03.055 29567554

[20] Park D, Middleton A, Smith R, Deblonde G, Laudal D, Theaker N, Hsu-Kim H, Jiao Y. 2020. A biosorption-based approach for selective extraction of rare earth elements from coal byproducts. Sep Purif Technol 241:116726. doi:10.1016/j.seppur.2020.116726

[21] Jiang N-J, Yoshioka H, Yamamoto K, Soga K. 2016. Ureolytic activities of a urease-producing bacterium and purified urease enzyme in the anoxic condition: implication for subseafloor sand production control by microbially induced carbonate precipitation (MICP). Ecol Eng 90:96–104. doi:10.1016/j.ecoleng.2016.01.073

[22] Fan Y, Hu X, Zhao Y, Wu M, Wang S, Wang P, Xue Y, Zhu S. 2020. Urease producing microorganisms for coal dust suppression isolated from coal: characterization and comparative study. Adv Powder Technol 31:4095–4106. doi:10.1016/j.apt.2020.08.014

[23] Abo-El-Enein SA, Ali AH, Talkhan FN, Abdel-Gawwad HA. 2012. Utilization of microbial induced calcite precipitation for sand consolidation and mortar crack remediation. HBRC J 8:185–192. doi:10.1016/j.hbrcj.2013.02.001

[24] Carter MS, Tuttle MJ, Mancini JA, Martineau R, Hung C-S, Gupta MK. 2023. Microbially induced calcium carbonate precipitation by Sporosarcina pasteurii: a case study in optimizing biological CaCO_3_ precipitation. Appl Environ Microbiol 89:e0179422. doi:10.1128/aem.01794-22 37439668 PMC10467343

[25] Hammes F, Verstraete* W. 2002. Key roles of pH and calcium metabolism in microbial carbonate precipitation. Rev Environ Sci Biotechnol 1:3–7. doi:10.1023/A:1015135629155

[26] Qian C, Ren X, Rui Y, Wang K. 2021. Characteristics of bio-CaCO_3_ from microbial bio-mineralization with different bacteria species. Biochem Eng J 176:108180. doi:10.1016/j.bej.2021.108180

[27] Pei D, Liu Z-M, Hu B-R, Wu W-J. 2020. Progress on mineralization mechanism and application research of Sporosarcina pasteurii. Prog Biochem Biophys 47:467–482. doi:10.16476/j.pibb.2020.0012

[28] Técher D, Grosjean N, Sohm B, Blaudez D, Le Jean M. 2020. Not merely noxious? Time-dependent hormesis and differential toxic effects systematically induced by rare earth elements in Escherichia coli. Environ Sci Pollut Res Int 27:5640–5649. doi:10.1007/s11356-019-07002-z 31845278

[29] Hoffmann TD, Reeksting BJ, Gebhard S. 2021. Bacteria-induced mineral precipitation: a mechanistic review. Microbiology (Reading) 167:1049. doi:10.1099/mic.0.001049 PMC8289221 33881981

[30] Fan J, Onal Okyay T, Frigi Rodrigues D. 2014. The synergism of temperature, pH and growth phases on heavy metal biosorption by two environmental isolates. J Hazard Mater 279:236–243. doi:10.1016/j.jhazmat.2014.07.016 25064261

[31] Li M, Cheng X, Guo H. 2013. Heavy metal removal by biomineralization of urease producing bacteria isolated from soil. Int Biodeterior Biodegrad 76:81–85. doi:10.1016/j.ibiod.2012.06.016

[32] Dhami NK, Quirin MEC, Mukherjee A. 2017. Carbonate biomineralization and heavy metal remediation by calcifying fungi isolated from karstic caves. Ecol Eng 103:106–117. doi:10.1016/j.ecoleng.2017.03.007

[33] Hu X, Su J, Ali A, Wang Z, Wu Z. 2021. Heterotrophic nitrification and biomineralization potential of Pseudomonas sp. HXF1 for the simultaneous removal of ammonia nitrogen and fluoride from groundwater. Bioresour Technol 323:124608. doi:10.1016/j.biortech.2020.124608 33421833

[34] Yi H, Zheng T, Jia Z, Su T, Wang C. 2021. Study on the influencing factors and mechanism of calcium carbonate precipitation induced by urease bacteria. J Cryst Growth 564:126113. doi:10.1016/j.jcrysgro.2021.126113

[35] Costa T da, Silva M da, Vieira MGA. 2020. Recovery of rare-earth metals from aqueous solutions by bio/adsorption using non-conventional materials: a review with recent studies and promising approaches in column applications. J Rare Earths 38:339–355. doi:10.1016/j.jre.2019.06.001

[36] Arul Manikandan N, Lens PNL. 2022. Biorefining of green macroalgal (Ulva sp.) biomass and its application in the adsorptive recovery of rare earth elements (REEs). Sep Purif Technol 303:122200. doi:10.1016/j.seppur.2022.122200

[37] Bergsten-Torralba LR, Nascimento CRS, Buss DF, Giese EC. 2021. Kinetics and equilibrium study for the biosorption of lanthanum by Penicillium simplicissimum INCQS 40,211. 3 Biotech 11:460. doi:10.1007/s13205-021-03004-2 PMC8519981 34722100

[38] Wang Z, Su J, Ali A, Yang W, Zhang R, Li Y, Zhang L, Li J. 2022. Chitosan and carboxymethyl chitosan mimic biomineralization and promote microbially induced calcium precipitation. Carbohydr Polym 287:119335. doi:10.1016/j.carbpol.2022.119335 35422299

[39] Doyle RJ, Matthews TH, Streips UN. 1980. Chemical basis for selectivity of metal ions by the Bacillus subtilis cell wall. J Bacteriol 143:471–480. doi:10.1128/jb.143.1.471-480.1980 6772632 PMC294273

[40] Zheng X, Hu P, Yao R, Cheng J, Chang Y, Mei H, Sun S, Chen S, Wen H. 2022. Biosorption behavior and biomineralization mechanism of low concentration uranium (VI) by Pseudomonas fluorescens. J Radioanal Nucl Chem 331:4675–4684. doi:10.1007/s10967-022-08551-3

[41] Kang X, Csetenyi L, Gadd GM. 2019. Biotransformation of lanthanum by Aspergillus niger. Appl Microbiol Biotechnol 103:981–993. doi:10.1007/s00253-018-9489-0 30443797 PMC6373195

[42] Kang X, Csetenyi L, Gadd GM. 2020. Monazite transformation into Ce- and La-containing oxalates by Aspergillus niger. Environ Microbiol 22:1635–1648. doi:10.1111/1462-2920.14964 32114711

[43] Yang W, Xu L, Wang Z, Li K, Hu R, Su J, Zhang L. 2022. Synchronous removal of ammonia nitrogen, phosphate, and calcium by heterotrophic nitrifying strain Pseudomonas sp. Y1 based on microbial induced calcium precipitation. Bioresour Technol 363:127996. doi:10.1016/j.biortech.2022.127996 36150425

[44] Duan Y, Xu G, Yang D, Yan Y. 2019. Influencing factors of calcium ion utilization in MICP mineralized products and analysis of microscopic image. Chem Ind Engeering Prog 38:2306–2313. doi:10.16085/j.issn.1000-6613.272018-1537

[45] Huang L, Yu Q, Liu W, Wang J, Guo W, Jia E, Zeng Q, Qin R, Zheng J, Hofmockel KS, Dong H, Jiang H, Zhu Z. 2021. Molecular determination of organic adsorption sites on Smectite during Fe redox processes using TOF-SIMS analysis. Environ Sci Technol 55:7123–7134. doi:10.1021/acs.est.0c08407 33901397

[46] Cui M-J, Teng A, Chu J, Cao B. 2022. A quantitative, high-throughput urease activity assay for comparison and rapid screening of ureolytic bacteria. Environ Res 208:112738. doi:10.1016/j.envres.2022.112738 35041816

[47] Omoregie AI, Khoshdelnezamiha G, Senian N, Ong DEL, Nissom PM. 2017. Experimental optimisation of various cultural conditions on urease activity for isolated Sporosarcina pasteurii strains and evaluation of their biocement potentials. Ecol Eng 109:65–75. doi:10.1016/j.ecoleng.2017.09.012

[48] Zeng Y, Chen Z, Du Y, Wang X, Zhou S, Xu L, Jiang X, Liu Y, Q, LÜ, Yan Z. 2022. The mineralization study of Pb (II), Cd (II) and Cr (VI) by induced calcite precipitation by urease producing strain Sporosarcina ureilytica ML-2. Acta Scientiae Circumstantiae 42:148–159. doi:10.13671/j.hjkxxb.2021.0450

[49] Sun Y, Su J, Ali A, Wang Z, Zhang S, Zheng Z, Min Y. 2022. Fungal-sponge composite carriers coupled with denitrification and biomineralization bacteria to remove nitrate, calcium, and cadmium in a bioreactor. Bioresour Technol 355:127259. doi:10.1016/j.biortech.2022.127259 35550924

